# Supplementary material for: Analysis of PPARγ Signaling Activity in Psoriasis
Source: Int J Mol Sci. 2021 Aug 10;22(16):8603. doi: 10.3390/ijms22168603 (PMC8395241; doi:10.3390/ijms22168603)
Supplement: Supplementary file 1 [file ijms-22-08603-s001.zip › Supplemental materials_Analysis of PPARg signaling activity in psoriasis/Pathway models/Models images and html files/Differentiation of psoriatic T cells/37672.html]

IL22


# Protein IL22

|  |  |
| --- | --- |
| URN | urn:agi-llid:50616 |
| Total Entities | 0 |
| Connectivity | 2923 |
| Name | IL22 |
| Description | interleukin 22 |
| Notes | This gene is a member of the IL10 family of cytokines that mediate cellular inflammatory responses. The encoded protein functions in antimicrobial defense at mucosal surfaces and in tissue repair. This protein also has pro-inflammatory properties and plays a role in in the pathogenesis of several intestinal diseases. [provided by RefSeq, Jul 2018] |
| Primary Cell Localization | Extracellular |
| Class | Ligand |

---

|  |  |
| --- | --- |
| Pathway | AHR Signaling in Th17 Cells Function |
|  | Th17-Cell Differentiation |
|  | AHR in Intestinal Cell Antimicrobial Barrier Maintenance |
|  | Proteins Involved in Myocarditis |
|  | Proteins Involved in Acne Vulgaris |
|  | Proteins Involved in Psoriasis |
|  | T-Cells Differentiation Block in Psoriasis |
|  | IL22 Induces Keratinocyte Proliferation in Psoriasis |
|  | Proteins Involved in Vitiligo |
|  | Th17-Cell Activation in Crohn's Disease |
|  | Proteins Involved in Inflammatory Bowel Diseases |
|  | Proteins Involved in Ulcerative Colitis |
|  | Th17-Cell Differentiation in Asthma |
|  | Th17-Cell and Th1 Immune Responsein Psoriatic Arthritis |
|  | Keratinocyte Activation in Psoriatic Arthritis |
|  | Th17-Cell Numbers Reduction in HIV |
|  | Proteins with Altered Expression in Psoriatic Arthritis |
|  | Proteins Involved in Age-Related Macular Degeneration |
|  | Elevated Receptors -> Expression Targets in Bone and Joint |
|  | STAT3 and NFkB Activate Inflammation-Induced Tumorigenesis |
|  | Proteins with Altered Expression in Tumor-Promoting Inflammation |
|  | NOTCH -> Expression Targets in Skin |
|  | IL22R -> STAT3 Signaling |
|  | IL22 Expression Targets |
|  | Interleukin Receptors -> Expression Targets in Lymphoid System and Blood |
|  | NOTCH -> Expression Targets in Thymus |
|  | PPAR Psoriasis |
|  | pprarg neg, uknown targets, ps-positive |
|  | prarg neg,ukn expres targets, ps-positive |
|  | PPARG negative targets |
|  | pparg expr target\_regulators |
|  | Neighbors of disease exacerbation |
|  | Neighbors of tumor progression |
|  | Neighbors of cancer progression |
|  | Neighbors of inflammatory status |
|  | Neighbors of overall survival |
|  | Neighbors of disease severity |
|  | Neighbors of tumor size |
|  | Neighbors of mortality |
|  | Neighbors of survival rate |
|  | Neighbors of functional recovery |
|  | Neighbors of insulin sensitivity |
|  | Neighbors of bacterial load |
|  | Neighbors of cancer survival |
|  | Neighbors of graft survival |
|  | Neighbors of therapeutic efficacy |
|  | Neighbors of lethality |
|  | Neighbors of treatment outcome |
|  | Neighbors of body weight |
|  | Neighbors of body weight gain |
|  | Neighbors of cardiovascular risk |
|  | Neighbors of glucose tolerance |
|  | Neighbors of morbidity |
|  | Neighbors of fibrosis progression |
|  | Neighbors of mortality rate |
|  | Neighbors of fat mass |
|  | Neighbors of viral load |
|  | Neighbors of blood pressure |
|  | Neighbors of tumor grade |
|  | Neighbors of blood glucose |
|  | Neighbors of T-cell count |
|  | Neighbors of cell permeability |
|  | Neighbors of pregnancy outcome |
|  | Neighbors of functional status |
|  | Neighbors of skin thickness |
|  | Neighbors of comorbidity |
|  | Neighbors of fibroblast phenotype |
|  | Neighbors of disease exacerbation |
|  | Neighbors of tumor progression |
|  | Neighbors of cancer progression |
|  | Neighbors of inflammatory status |
|  | Neighbors of overall survival |
|  | Neighbors of disease severity |
|  | Neighbors of tumor size |
|  | Neighbors of mortality |
|  | Neighbors of survival rate |
|  | Neighbors of functional recovery |
|  | Neighbors of insulin sensitivity |
|  | Neighbors of bacterial load |
|  | Neighbors of cancer survival |
|  | Neighbors of graft survival |
|  | Neighbors of therapeutic efficacy |
|  | Neighbors of lethality |
|  | Neighbors of treatment outcome |
|  | Neighbors of body weight |
|  | Neighbors of body weight gain |
|  | Neighbors of cardiovascular risk |
|  | Neighbors of glucose tolerance |
|  | Neighbors of morbidity |
|  | Neighbors of fibrosis progression |
|  | Neighbors of mortality rate |
|  | Neighbors of fat mass |
|  | Neighbors of viral load |
|  | Neighbors of blood pressure |
|  | Neighbors of tumor grade |
|  | Neighbors of blood glucose |
|  | Neighbors of T-cell count |
|  | Neighbors of cell permeability |
|  | Neighbors of pregnancy outcome |
|  | Neighbors of functional status |
|  | Neighbors of skin thickness |
|  | Neighbors of comorbidity |
|  | Neighbors of fibroblast phenotype |
|  | Neighbors of cell development |
|  | Neighbors of immune response |
|  | Neighbors of cell population |
|  | Neighbors of T-cell development |
|  | Neighbors of inflammatory response |
|  | Neighbors of wound healing |
|  | Neighbors of angiogenesis |
|  | Neighbors of innate immune response |
|  | Neighbors of cell function |
|  | Neighbors of macrophage activation |
|  | Neighbors of cell formation |
|  | Neighbors of adaptive immune response |
|  | Neighbors of osteoclast development |
|  | Neighbors of epithelial to mesenchymal transition |
|  | Neighbors of immunity |
|  | Neighbors of cell proliferative response |
|  | Neighbors of chemotaxis |
|  | Neighbors of cell survival |
|  | Neighbors of osteoclast differentiation |
|  | Neighbors of cell phenotype |
|  | Neighbors of T-cell proliferation |
|  | Neighbors of tissue remodeling |
|  | Neighbors of cell adhesion |
|  | Neighbors of endothelial cell proliferation |
|  | Neighbors of fibrogenesis |
|  | Neighbors of tumor growth |
|  | Neighbors of tissue repair |
|  | Neighbors of cell count |
|  | Neighbors of cell infiltration |
|  | Neighbors of cell interaction |
|  | Neighbors of SMC proliferation |
|  | Neighbors of lumen formation |
|  | Neighbors of endothelial cell migration |
|  | Neighbors of cell damage |
|  | Neighbors of pregnancy |
|  | Neighbors of cell motility |
|  | Neighbors of fibroblast proliferation |
|  | Neighbors of neutrophil recruitment |
|  | Neighbors of regeneration |
|  | Neighbors of cell migration |
|  | Neighbors of cancer growth |
|  | Neighbors of stem cell proliferation |
|  | Neighbors of smooth muscle cell migration |
|  | Neighbors of hepatic regeneration |
|  | Neighbors of phagocytosis |
|  | Neighbors of leukocyte migration |
|  | Neighbors of leukocyte recruitment |
|  | Neighbors of osteoclast formation |
|  | Neighbors of cell invasion |
|  | Neighbors of heart function |
|  | Neighbors of bone remodeling |
|  | Neighbors of ossification |
|  | Neighbors of T-cell function |
|  | Neighbors of immune system function |
|  | Neighbors of autophagy |
|  | Neighbors of transcription activation |
|  | Neighbors of senescence |
|  | Neighbors of T-cell response |
|  | Neighbors of tumor immunity |
|  | Neighbors of hepatocyte proliferation |
|  | Neighbors of cell growth |
|  | Neighbors of ROS generation |
|  | Neighbors of epithelial cell proliferation |
|  | Neighbors of cancer cell growth |
|  | Neighbors of sensitization |
|  | Neighbors of hemato-encephalic barrier |
|  | Neighbors of cellular immune response |
|  | Neighbors of cellular senescence |
|  | Neighbors of colony formation |
|  | Neighbors of cell differentiation |
|  | Neighbors of keratinocyte proliferation |
|  | Neighbors of cell homeostasis |
|  | Neighbors of psoriasis |
|  | Neighbors of inflammatory disease |
|  | Neighbors of rheumatoid arthritis |
|  | Neighbors of atherosclerosis |
|  | Neighbors of fibrosis |
|  | Neighbors of colitis |
|  | Neighbors of arthritis |
|  | Neighbors of inflammatory bowel disease |
|  | Neighbors of injury |
|  | Neighbors of atherogenesis |
|  | Neighbors of vascular remodeling |
|  | Neighbors of metastasis |
|  | Neighbors of carcinogenesis |
|  | Neighbors of multiple sclerosis |
|  | Neighbors of chronic inflammation |
|  | Neighbors of inflammation |
|  | Neighbors of pathological angiogenesis |
|  | Neighbors of obesity |
|  | Neighbors of diabetes mellitus |
|  | Neighbors of asthma |
|  | Neighbors of pneumonia |
|  | Neighbors of hyperplasia |
|  | Neighbors of osteoarthritis |
|  | Neighbors of liver fibrosis |
|  | Neighbors of chronic obstructive pulmonary disease |
|  | Neighbors of myocardial infarction |
|  | Neighbors of leukocyte infiltration |
|  | Neighbors of reperfusion injury |
|  | Neighbors of airway inflammation |
|  | Neighbors of hepatitis |
|  | Neighbors of insulin resistance |
|  | Neighbors of infection |
|  | Neighbors of renal injury |
|  | Neighbors of liver injury |
|  | Neighbors of sepsis |
|  | Neighbors of death |
|  | Neighbors of type 1 diabetes |
|  | Neighbors of encephalomyelitis |
|  | Neighbors of autoimmune disease |
|  | Neighbors of dermatitis |
|  | Neighbors of neutrophil infiltration |
|  | Neighbors of lung injury |
|  | Neighbors of immunopathology |
|  | Neighbors of systemic sclerosis |
|  | Neighbors of hepatocellular carcinoma |
|  | Neighbors of autoimmunity |
|  | Neighbors of acute lung injury |
|  | Neighbors of gastric cancer |
|  | Neighbors of experimental autoimmune encephalomyelitis |
|  | Neighbors of colorectal cancer |
|  | Neighbors of ischemia |
|  | Neighbors of systemic lupus erythematosus |
|  | Neighbors of collagen-induced arthritis |
|  | Neighbors of interstitial fibrosis |
|  | Neighbors of kidney disease |
|  | Neighbors of heart failure |
|  | Neighbors of lung disease |
|  | Neighbors of pulmonary fibrosis |
|  | Neighbors of cancer |
|  | Neighbors of type 2 diabetes |
|  | Neighbors of neoplasm |
|  | Neighbors of vasculitis |
|  | Neighbors of atrophy |
|  | Neighbors of diet-induced obesity |
|  | Neighbors of respiratory hypersensitivity |
|  | Neighbors of myocardial fibrosis |
|  | Neighbors of synovitis |
|  | Neighbors of coronary artery disease |
|  | Neighbors of endothelial cell dysfunction |
|  | Neighbors of liver disease |
|  | Neighbors of hypertrophy |
|  | Neighbors of pancreatic cancer |
|  | Neighbors of hypertension |
|  | Neighbors of allograft rejection |
|  | Neighbors of epithelial cell |
|  | Neighbors of keratinocyte |
|  | Neighbors of umbilical vein endothelial cell |
|  | Neighbors of monocyte |
|  | Neighbors of endometrial stromal cell |
|  | Neighbors of alveolar macrophage |
|  | Neighbors of macrophage |
|  | Neighbors of fibroblast |
|  | Neighbors of PBMC |
|  | Neighbors of pneumocyte |
|  | Neighbors of intestine epithelium cell |
|  | Neighbors of mononuclear cell |
|  | Neighbors of hepatocyte |
|  | Neighbors of decidual cell |
|  | Neighbors of endothelial cell |
|  | Neighbors of dendritic cell |
|  | Neighbors of inflammatory cell |
|  | Neighbors of neutrophil |
|  | Neighbors of bone marrow derived dendritic cell |
|  | Neighbors of activated T-cell |
|  | Neighbors of mononuclear phagocyte |
|  | Neighbors of immunocompetent cell |
|  | Neighbors of granulocyte |
|  | Neighbors of cancer-associated fibroblast |
|  | Neighbors of stem cell |
|  | Neighbors of B-cell |
|  | Neighbors of leukocyte |
|  | Neighbors of T-cell |
|  | Neighbors of M1 macrophage |
|  | Neighbors of mast cell |
|  | Neighbors of spleen cell |
|  | Neighbors of hematopoietic cell |
|  | Neighbors of antigen-presenting cell |
|  | Neighbors of epidermis cell |
|  | Neighbors of phagocyte |
|  | Neighbors of kidney cell |
|  | Neighbors of lymphocyte |
|  | Neighbors of Langerhans cell |
|  | Neighbors of decidual natural killer cell |
|  | Neighbors of lymphoid cell |
|  | Neighbors of blood cell |
|  | Neighbors of skin cell |
|  | Neighbors of lymph node cell |
|  | Neighbors of lung |
|  | Neighbors of liver |
|  | Neighbors of kidney |
|  | Neighbors of skin |
|  | Neighbors of intestine |
|  | Neighbors of colon |
|  | Neighbors of airway |
|  | Neighbors of gastrointestinal tract |
|  | Neighbors of bone |
|  | Neighbors of synovial membrane |
|  | Neighbors of islets of Langerhans |
|  | Neighbors of joint |
|  | Neighbors of cardiovascular system |
|  | Neighbors of lymphatic system |
|  | Neighbors of spleen |
|  | Neighbors of lymph node of mesentery |
|  | Neighbors of myelin sheath |
|  | Neighbors of draining lymph node |
|  | Neighbors of mesentery |
|  | Neighbors of salivary gland |
|  | Neighbors of ileum |
|  | Neighbors of small intestine |
|  | Neighbors of NF-kB family |
|  | Neighbors of IL1 family |
|  | Neighbors of PPAR |
|  | Neighbors of Jun/Fos |
|  | Neighbors of PI3K |
|  | Neighbors of IFNAR ligand |
|  | Neighbors of transforming growth factor |
|  | Neighbors of TLR |
|  | Neighbors of cytokine |
|  | Neighbors of interleukin |
|  | Neighbors of MEK1/2 |
|  | Neighbors of Ras GTPase |
|  | Neighbors of PKA |
|  | Neighbors of NF-AT family |
|  | Neighbors of LXR |
|  | Neighbors of IL23 |
|  | Neighbors of T-cell receptor |
|  | Neighbors of IL1R |
|  | Neighbors of IL12 |
|  | Neighbors of Notch |
|  | Neighbors of lectin |
|  | Neighbors of interleukin-35 |
|  | Neighbors of class nuclear receptor with C4 zinc fingers |
|  | Neighbors of immunoglobulin |
|  | Neighbors of CD3 complex |
|  | Neighbors of RA receptor |
|  | comon ps\_pos, pprarg\_neg targets |
|  | Model of PPARG signaling in psoriasis |
|  | PPARG negative regulators and targets |
|  | Model of PPARG related pathways in psoriasis (short version) |
|  | Overview. Diseases of the skin (chapter 11) |
|  | 1\_Differentiation of psoriatic T cells |
|  | 2\_Interleukin-17 and interleukin-22 signaling in psoriasis |
|  | 4\_1\_Polymorphisms associated with inflammatory bowel diseases |
|  | 1\_Inflammatory mediators increase the production of hepcidin (HAMP) |
|  | Model of PPARG signaling in psoriais (tested) |
|  | Differentiation of psoriatic T cells |
|  | Interleukin-17 and interleukin-22 signaling in psoriasis |

---

|  |  |
| --- | --- |
| MedScan ID | 50616 |

---

|  |  |
| --- | --- |
| LocusLink ID | 50616 |
|  | 50929 |
|  | 116849 |
|  | 500836 |

---

|  |  |
| --- | --- |
| Alias | interleukin-22 receptor ligand |
|  | IL-TIFbs |
|  | interleukin IL-TIF |
|  | interleukin IL-TIFb |
|  | interleukin IL-10-related T-cell-derived inducible factor |
|  | TIFIL-23 |
|  | IL-10-related T-cell-derived-inducible factor beta |
|  | ILTIF |
|  | ILTIFa |
|  | MGC79382 |
|  | interleukin-22b |
|  | ILTIF alpha |
|  | RGD1561292 |
|  | MGC129416 |
|  | LOC236012 |
|  | cytokine Zcyto18 |
|  | interleukin-22 receptor ligand activity |
|  | mCG\_123110 |
|  | UNQ3099/PRO10096 |
|  | MGC129417 |
|  | zcyto18 |
|  | IL-TIF alpha |
|  | LOC236571 |
|  | interleukin IL-10-related T-cell-derived inducible factor alpha |
|  | ILTIFb |
|  | interleukin IL-D110 |
|  | IL-TIF beta |
|  | T-cell-derived inducible factor alpha |
|  | MGC79384 |
|  | IL-TIF |
|  | interleukin 10-related T cell-derived inducible factor beta |
|  | IL-TIFa |
|  | interleukin IL-TIFa |
|  | interleukin-22 receptor ligands |
|  | IL-22a |
|  | interleukin IL22 |
|  | interleukin 10-related T cell-derived inducible factor |
|  | interleukin 22 |
|  | Interleukin-22a precursor (IL-22a) (IL-10-related T-cell-derived inducible factor alpha) (IL-TIFa) |
|  | IL22 |
|  | interleukin IL-TIFbs |
|  | IL-D110 |
|  | IL-10-related T-cell-derived inducible factor alpha |
|  | IL-10-related T-cell-derived inducible factor |
|  | IL-TIFb |
|  | interleukin-22a |
|  | Il22b |
|  | T-cell-derived inducible factor beta |
|  | TIFa |
|  | IL-21 |
|  | IL-22 |
|  | interleukin-22 |
|  | IL-10-related T-cell-derived-inducible factor |
|  | IL-22b |
|  | IL21 |
|  | interleukin 21 |

---

|  |  |
| --- | --- |
| GO ID | 0005125 |
|  | 0045518 |
|  | 0006953 |
|  | 0019221 |
|  | 0006954 |
|  | 0051384 |
|  | 0005576 |
|  | 0005615 |
|  | 0050728 |
|  | 0045944 |
|  | 0072593 |
|  | 0042509 |
|  | 0010469 |
|  | 0007267 |
|  | 0006955 |
|  | 0046427 |
|  | 0042516 |

---

|  |  |
| --- | --- |
| KEGG ID | mmu:50929 |
|  | mmu:116849 |
|  | hsa:50616 |
|  | rno:500836 |

---

|  |  |
| --- | --- |
| Organism | Homo sapiens {Organism urn:agi-taxid:9606} |
|  | Mus musculus {Organism urn:agi-taxid:10090} |
|  | Rattus norvegicus {Organism urn:agi-taxid:10116} |
|  | Mus musculus |
|  | Homo sapiens |
|  | Rattus norvegicus |

---

|  |  |
| --- | --- |
| Mouse chromosome position | 10 66.67 cM |
|  | 10 |
|  | 10 67.0 cM |

---

|  |  |
| --- | --- |
| OMIM ID | 605330 |

---

|  |  |
| --- | --- |
| Rat chromosome position | 7q22 |

---

|  |  |
| --- | --- |
| Hugo ID | 14900 |
|  | HGNC:14900 |

---

|  |  |
| --- | --- |
| Human chromosome position | 12q15 |

---

|  |  |
| --- | --- |
| Swiss-Prot Accession | Q9GZX6 |
|  | Q9GZX6.1 |
|  | Q9JJY9 |
|  | Q9JJY9.1 |
|  | Q9JJY8 |
|  | Q9JJY8.2 |
|  | G3V6X6 |
|  | Q14BB3 |
|  | E9QAL9 |
|  | Q6NWP4 |
|  | Q6NZ83 |
|  | Q6NZ84 |
|  | Q3SXE1 |
|  | Q198B4 |

---

|  |  |
| --- | --- |
| GenBank ID | NC\_000012 |
|  | NM\_020525 |
|  | NP\_065386 |
|  | NG\_060763 |
|  | AC007458 |
|  | AC022511 |
|  | AF387519 |
|  | AAK62468 |
|  | AJ277248 |
|  | CAC19409 |
|  | AX151713 |
|  | CAC42593 |
|  | AX179578 |
|  | CAC49973 |
|  | AX468783 |
|  | CAD42564 |
|  | CH471054 |
|  | EAW97182 |
|  | CQ889879 |
|  | CAH61624 |
|  | CQ889941 |
|  | CAH61629 |
|  | CS425140 |
|  | CAL60104 |
|  | CS719533 |
|  | CAP40185 |
|  | HC679523 |
|  | CBL86572 |
|  | HC888910 |
|  | CBM42922 |
|  | JA104587 |
|  | CCA63963 |
|  | AF279437 |
|  | AAG22064 |
|  | AJ277247 |
|  | CAC06085 |
|  | AY358890 |
|  | AAQ89249 |
|  | BC066263 |
|  | AAH66263 |
|  | BC066264 |
|  | AAH66264 |
|  | BC066265 |
|  | AAH66265 |
|  | BC066266 |
|  | BC067509 |
|  | AAH67509 |
|  | BC067510 |
|  | AAH67510 |
|  | BC069112 |
|  | AAH69112 |
|  | BC069308 |
|  | AAH69308 |
|  | BC070261 |
|  | AAH70261 |
|  | CD559617 |
|  | CD638517 |
|  | Q9GZX6 |
|  | NC\_000076 |
|  | XM\_006513865 |
|  | XP\_006513928 |
|  | NM\_016971 |
|  | NP\_058667 |
|  | AC153856 |
|  | AJ294727 |
|  | CAC19435 |
|  | CH466539 |
|  | EDL21856 |
|  | AJ249491 |
|  | CAB75546 |
|  | BC104348 |
|  | AAI04349 |
|  | BC104349 |
|  | AAI04350 |
|  | BC116235 |
|  | AAI16236 |
|  | Q9JJY9 |
|  | NM\_054079 |
|  | NP\_473420 |
|  | AC158600 |
|  | AJ294728 |
|  | CAC19436 |
|  | AJ249492 |
|  | CAB75547 |
|  | AK005228 |
|  | Q9JJY8 |
|  | NC\_005106 |
|  | NM\_001191988 |
|  | NP\_001178917 |
|  | AC\_000075 |
|  | AABR07057220 |
|  | AAHX01049469 |
|  | AAHX01049470 |
|  | CH473960 |
|  | EDM16599 |
|  | DQ630444 |
|  | ABF82262 |
|  | NC\_018923 |
|  | AMYH02026693 |
|  | HQ448427 |
|  | ADQ32907 |
|  | AC\_000032 |
|  | AAHY01089284 |
|  | NW\_001074832 |
|  | AAHY01146294 |
|  | AC\_000144 |
|  | NT\_029419 |
|  | NW\_001838060 |
|  | NW\_925395 |
|  | AC\_000055 |
|  | Q6NWP4 |
|  | Q6NZ83 |
|  | Q6NZ84 |
|  | NT\_039500 |
|  | NW\_001030420 |
|  | Q3SXE1 |
|  | XM\_576228 |
|  | XP\_576228 |
|  | NW\_001084856 |
|  | XM\_001081002 |
|  | XP\_001081002 |
|  | NW\_047774 |
|  | Q198B4 |
|  | XM\_005268929 |
|  | XP\_005268986 |
|  | ABBA01026559 |

---

|  |  |
| --- | --- |
| Swiss-Prot ID | IL22\_MOUSE |
|  | IL22B\_MOUSE |
|  | IL22\_HUMAN |
|  | E9QAL9\_MOUSE |
|  | Q14BB3\_MOUSE |

---

|  |  |
| --- | --- |
| Cell Localization | Secreted |
|  | Extracellular |

---

|  |  |
| --- | --- |
| Ensembl ID | ENSG00000127318 |
|  | ENSP00000329384.4 |
|  | ENST00000328087.6 |
|  | ENSMUSG00000074695 |
|  | ENSMUSP00000094449.4 |
|  | ENSMUST00000096691.4 |
|  | ENSMUSG00000090461 |
|  | ENSMUSP00000128415.1 |
|  | ENSMUST00000163808.1 |
|  | ENSRNOG00000007365 |
|  | ENSRNOP00000009776.3 |
|  | ENSRNOT00000009776.4 |
|  | ENST00000328087.5 |
|  | ENSP00000329384 |
|  | ENST00000328087 |
|  | ENSMUSP00000094449 |
|  | ENSMUST00000096691 |
|  | ENSMUSP00000128415 |
|  | ENSMUST00000163808 |
|  | ENSRNOP00000009776 |
|  | ENSRNOT00000009776 |
|  | ENSP00000442424 |
|  | ENST00000538666 |

---

|  |  |
| --- | --- |
| MGI ID | MGI:1355307 |
|  | MGI:2151139 |
|  | 1355307 |
|  | 2151139 |

---

|  |  |
| --- | --- |
| RGD ID | 1561292 |

---

|  |  |
| --- | --- |
| Unigene ID | Mm.103585 |
|  | Mm.213193 |
|  | Rn.195879 |
|  | Hs.287369 |

---

|  |  |
| --- | --- |
| Homologene ID | 9669 |

---

|  |  |
| --- | --- |
| Shape | Rhomb |

---

|  |  |
| --- | --- |
| IPI ID | IPI00008372 |
|  | IPI00122408 |
|  | IPI00953777 |
|  | IPI00122407 |
|  | IPI00769042 |
|  | IPI00475569 |

---
